# Clinical Evaluation of an Electronic Guidance System for Optimizing the Ultrasound Screening for Developmental Hip Dysplasia in Newborns

**DOI:** 10.3390/jcm13247656

**Published:** 2024-12-16

**Authors:** Stephan Heisinger, Catharina Chiari, Madeleine Willegger, Reinhard Windhager, Alexander Kolb

**Affiliations:** Department of Orthopedics and Trauma Surgery, Medical University of Vienna, 1090 Vienna, Austria; stephan.heisinger@meduniwien.ac.at (S.H.); madeleine.willegger@meduniwien.ac.at (M.W.);

**Keywords:** hip dysplasia, hip ultrasound, innovative 3D navigation, population health screening

## Abstract

**Background:** Graf ultrasound screening is considered an established method for early detection of developmental dysplasia of the hip (DDH). Although characterized by a high degree of standardization to allow for good reproducibility of results, examination-related factors may still affect sonographic measurements. The relative tilt angle between the hip and the probe is a potential pitfall as it significantly influences sonographic measurements and consequently classification of DDH according to Graf. **Objectives:** Evaluation of an electronic guidance system developed to reduce relative tilt angles and increase reliability and comparability in ultrasound screening of DDH. **Materials and Methods:** Twenty-five newborns were examined using a prototype guidance system, which tracks the position of the transducer and the pelvis to calculate the relative tilt angles. Two ultrasound images were obtained, one conventionally and the other one using the guidance system. Subsequently, relative roll and pitch angles and sonographic measurements were determined and analyzed. **Results:** The relative inclination angles in the conventional group ranged from −12.6° to 14.3° (frontal plane) and −23.8° to 32.5° (axial plane). vs. −3.7° to 3.0° and −3.2° to 4.5° in the guidance system group. The variances were significantly lower in the guidance system-assisted group for both planes (*p* < 0.001 and *p* < 0.001, respectively). The optimized transducer position showed significant effects and consequently significantly reduced alpha angles were observed (*p* = 0.001, and *p* = 0.003). **Conclusions:** The guidance system allowed a significant reduction in the relative tilt angles, supporting optimal positioning of the transducer, resulting in significant effects on Graf sonographic measurements. This technique shows great potential for enhancing the reproducibility and reliability of ultrasound screening for DDH.

## 1. Introduction

Developmental Dysplasia of the hip (DDH) is a developmental disorder that can result in various abnormalities of the hip joint such as neonatal instability of the hip joint, acetabular of femoral dysplasia, and hip subluxation and dislocation [1,2]. DDH may be isolated, but can also be found with concomitant defects such as clubfoot, torticollis, or other spine-related or neuromuscular disorders or diseases such as Down’s syndrome [3,4]. Tao et al. reported a pooled prevalence of DDH—resulting from 65 primary studies—to be 1.4%; however the studies showed great variances with regard to reported prevalence, which can be attributed to the varying regional incidence rates and screening programs [2,5]. As reviewed by Vaquero-Picado et al., risk factors for DDH are breech presentation, family history, female gender, oligohydramnios, elevated weight at birth, multiple pregnancy, left hip, hyperlaxity and clubfoot deformity [6]. As reviewed by Harsanyi et al., various genes and epigenetic modifications were found to be associated with DDH [7]. Graf ultrasound screening is widely considered the gold standard for early detection of developmental dysplasia of the hip in newborns [8,9,10]. This approach is defined by a high level of standardization in the sonographic examination, ensuring reliable reproducibility of results [11,12]. The classification is based on standardized measurement of the α-angle-angle between the bony acetabular roof and the vertical cortex of the ilium—and the β-angle-angle between the line drawn between the turning point and the center of the labrum—resembling the cartilage roof line—and the vertical cortex of the ilium [13]. While the α-angle is an indicator for bony femoral head coverage, the β-angle is used to evaluate femoral head cartilaginous coverage [2,13,14]. If DDH persists or is not detected early at all it results in altered biomechanics of the hip and overloading articular cartilage and consequently it may lead to early osteoarthritis of the hip [6]. DDH can be classified into four types according to Graf, where Type I resembles a normal hip (α > 60°, β < 55°), Type II (43° < α < 60°, 55°< β < 77°) a dysplastic hip or a hip with delayed ossification, Type III (α < 43°, β > 77°) resembles a subluxated hip, and Type IV (α < 43°, β cannot be measured) a dislocated hip [2,13].

Overall, it is estimated that up to 9.1% of all total hip replacement (THR) cases can be attributed to DDH, and it is the main cause of THR in young patients (approximately 21% to 29%) [6,15,16]. Taking this into consideration, the relevance of screening for DDH in regard to the potential socioeconomic burden is highlighted. Although clinical screening is widely recommended, there is no international consensus on hip ultrasound as a screening tool [17,18,19,20]. As reviewed by Kilsdonk et al., the lack of consensus leads to considerable variation in regard to screening programs across the world [17]. Overall there are few high-quality studies that compare the various screening programs; however, two studies from Austria and Germany were able to show a decrease in surgery rates and complications as well as a reduction in costs since the introduction of universal ultrasound screening [17,21,22]. Considering that the high efficiency of non-operative treatment when DDH is detected early, this highlights the relevance of screening programs, consequently reducing the rate of surgical interventions and the resulting socioeconomic burden [23].

Despite all of the efforts to standardize ultrasound screening for DDH, examination-related factors have been discussed that might affect sonographic measurements unfavorably and result in overestimated incidence rates of DDH [24]. When it comes to examination-related errors, the relative tilt angle between the hip being examined and the ultrasound probe is a critical factor, as shown by Jaremko et al. through the use of 3D ultrasound imaging [25]. So far, this issue has primarily been addressed using tools like a mechanical transducer guiding device and a baby positioning aid (cradle) [26].

An analysis of the effects of relative tilt angles between the hip joint and transducer on Graf’s method using an optoelectronic motion capture system demonstrated a highly significant effect on sonographic measurements and, furthermore, on Graf’s DDH classification [27]. However, the optoelectronic motion capture system used was unable to determine the position of the newborn’s hip or pelvis, which is a significant limitation since the position of the hip or pelvis is crucial for calculating the relative tilt angles [27]. To overcome this limitation, the development of the system used here was started [10].

This study aimed to assess the effectiveness of a new electronic navigation system in reducing examiner-related errors in the relative tilt angle between the ultrasound transducer and the examined hip joint. It also analyzed the system’s impact on sonographic measurements compared to traditional ultrasound screening based on Graf’s method.

## 2. Materials and Methods

The study received approval from the Institutional Review Board of the Medical University of Vienna (Ethical vote: EK-No: 1414/2012, dated 24 April 2015) and was conducted in compliance with applicable guidelines and regulations [10]. Informed consent was obtained from all legal guardians prior to participation.

**Figure 1:**
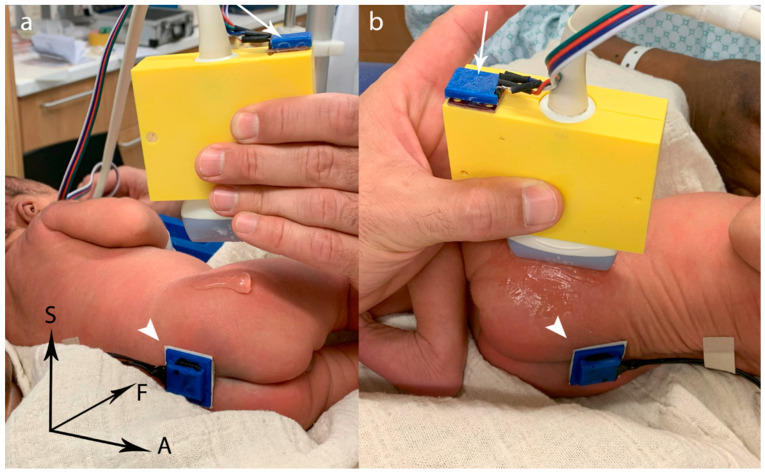
Illustration of the 3D position sensors: detection of the pelvic position by an epicutaneous sensor placed dorsally over the sacrum (arrowhead) and of the transducer position by a sensor fixed by a 3D printed adapter (thin arrow); (**a**) starting position: the coordination system is shown at the bottom left (normal vector of the frontal (F), axial (A) and sagittal (S) planes); (**b**) alignment of transducer and pelvis using the guidance system in the axial and frontal plane: note the tilted pelvic position and the different orientation rotation of the sensors in the sagittal plane.

An electronic guidance system was developed at our institution to determine relative tilt errors between the transducer and the hip joint under examination [10]. This electronic system utilizes two position sensors: one is mounted on the ultrasound transducer with a 3D-printed adapter, while the other is placed epicutaneously in a central position on the back, dorsal to the sacrum (see Figure 1) [10]. The two position sensors consist of a combination of accelerometer, gyroscope and magnetometer (MPU-9250, InvenSense Inc., San Jose, CA, USA) as described previously [10].

A specially developed software program calculates the relative inclination angles between the two sensors in the frontal, axial and sagittal planes in real time, representing roll, pitch and yaw angles, respectively (see Figure 1). These measurements are transmitted to PC-based output software that displays the angles in real time to assist navigation and stores the data. The measurement accuracy of the roll and pitch angles was established as ±1°, while the yaw angle accuracy was determined to be ±2°, as these accuracies were deemed clinically acceptable by the authors. A patent application has been submitted for this system [28].

In total, twenty-five consecutive newborns underwent sonographic screening within the first week of life by a single experienced examiner using the Graf’s method with a GE Logiq F8 series system with a 7.5 MHz linear transducer (GE Healthcare, Milwaukee, WI, USA) in 2021. In each child examined, two ultrasound images were obtained for each hip joint according to the following criteria: One ultrasound image was captured using the conventional method, following the sonographic criteria outlined by Graf [26] (group A). Another image was captured using the guidance system, complementing Graf’s sonographic criteria by optimizing the transducer’s position based on the calculated tilt angles (Group B) [10]. If a suitable image could not be acquired because of the newborn’s restlessness, the data collection process was halted.

### Statistical Analysis

Data were processed using SPSS Statistics 28 software (SPSS Inc., Chicago, IL, USA). Normal distribution of parameters was evaluated using Kolmogorov–Smirnov tests and Q-Q plots. The relative roll and pitch angles between the transducer and pelvic positions were evaluated using paired *t*-tests and Levene’s tests. The effects on sonographic α-angles were analyzed using paired *t*-tests. McNemar’s test was used to analyze the difference in classification according to Graf. The significance level was defined as *p* < 0.05.

## 3. Results

Out of the twenty-five newborns, nineteen had both hips measured following the study protocol, five had only one hip measured due to increased spontaneous movements during the examination, and one was excluded because of restlessness. Thus, sonographic images and relative 3D position data of 43 hips (86 measurements) were obtained according to the study protocol.

In group A (conventional sonography according to Graf) 39 hips were classified as Type I and 4 hips as Type II. In group B (guidance system enhanced sonography) 33 hips were classified as Type I and 10 hips as Type II. The observed difference in classification was found to be statistically significant (*p* = 0.031) No other types according to Graf were detected in our patient sample. Concludingly, the application of our guidance system resulted in an increased detection rate of Type II hips by reducing relative tilt angles.

## 4. Analysis of 3D-Data

**Figure 2:**
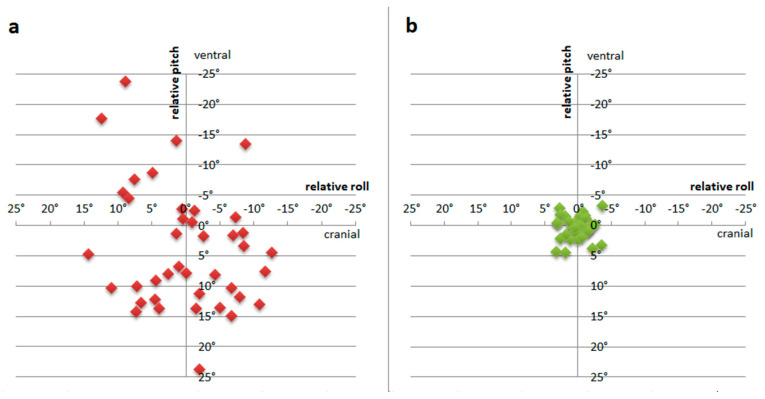
Relative roll and pitch angles: (**a**) conventional group according to Graf’s sonographic criteria [8], (**b**) guidance system assisted group in which the developed system was used to optimize relative transducer position in addition to Graf’s criteria [10].

In group A, where sonographic images were acquired without the guidance system, the relative tilt angles between the transducer and pelvis ranged from −12.6° to 14.3° for the roll angle (measured in the frontal plane) and from −23.8° to 32.5° for the pitch angle (measured in the axial plane). In group B, which used the guidance system, the relative tilt angles ranged from −3.7° to 3.0° in the frontal plane and −3.2° to 4.5° in the axial plane. (see Table 1). Comparison of variances of relative tilt angles showed highly significant differences with lower variance in group B (*p* < 0.001 for frontal and axial planes). In the axial plane a dorsal tilted transducer position was found more often in group A (mean pitch angle 4.4° versus 0.3° in group B; *p* = 0.022). In the frontal plane the tilt angles showed no significant differences between group A and B (0.2° and 0°; *p* = 0.854). Figure 2 shows the measured relative roll and pitch angles of both groups.

**Figure 3:**
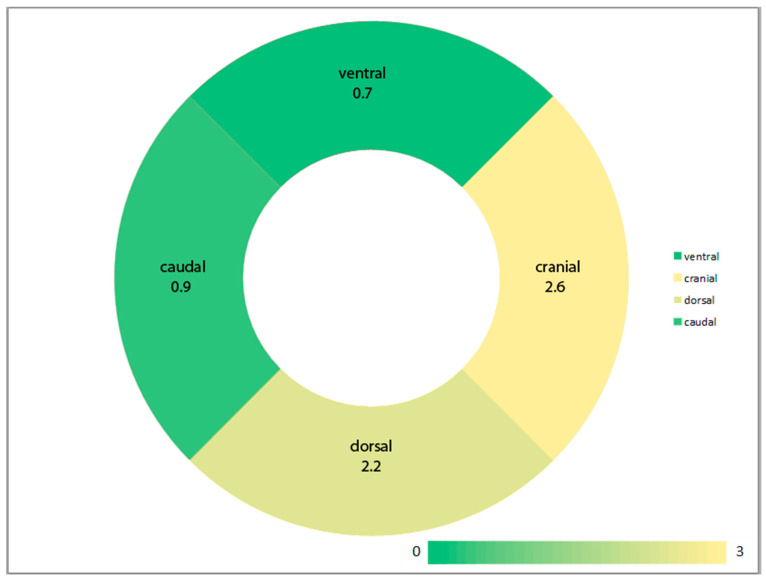
Illustration of the effects of correction of tilt angles on alpha angles. The average reduction in the alpha angle is color coded and inserted as a number.

Sonographic measurements according to Graf’s system showed slightly reduced alpha angles in group B (mean alpha angle 62.9° [SD 3.2] in group A and 61.3° [SD 3.5] in group B; *p* = 0.04). Correction of cranial and dorsal tilt errors using the guidance system showed significant effects on alpha angles (*p* = 0.001 and *p* = 0.003, respectively). The correction of caudal or ventral tilt angles showed no significant effects (*p* = 0.186 and *p* = 0.191). An overview of the effects of the tilt angle corrections is shown in Figure 3.

## 5. Discussion

The critical role of accurately positioning the ultrasound transducer relative to the pelvis in achieving reliable results during DDH ultrasound screening has been previously established [27,29]. Despite the standardization of Graf hip screening, a certain susceptibility of the method to relative tilt errors has been discussed [10,25,30]. The presence of unfavorable relative tilt angles and their significant effects on the obtained sonographic measurements was shown in an experimental study [27]. The importance of ensuring that the transducer is positioned in the correct plane was also demonstrated using 3D ultrasound imaging [25,30].

Since these studies primarily focused on achieving an optimal transducer position (i.e., measurement plane) without accounting for the pelvic position, the presented system was developed [10]. Given the varying spontaneous pelvic positions observed in clinical practice, this is an important step in overcoming a major limitation. In our previous study, we were able to show that the guidance system can significantly reduce the variances in relative tilt angles which we aimed to further evaluate in a larger sample [10].

Analysis of the 3D data in this study showed a significant reduction in the variance of the relative tilt angles between the pelvis and the transducer in the frontal and axial planes, confirming the findings of our previous study [10]. However, the aim of the present study was to provide initial insights into the effects of correcting tilt angles on sonographically measured alpha angles. The comparison between group A (conventional) and group B (guidance system assisted) showed that the correction of cranial tilt angles leads to reduced alpha angles. Interestingly, these cranial tilt angles are frequently combined with dorsal tilt angles, a combination which was reported to overestimate alpha angles [27]. Our findings support this relationship and underscore the importance of avoiding combined cranial/dorsal tilt errors. Surprisingly, the effect of caudal tilt errors was not significant. Here, we would expect that caudal tilt errors tend to underestimate the alpha angles [27]. However, this might be due to the experience of the examiner avoiding extensive caudal tilt errors in most cases (compare Figure 3 and Table 1). Beyond all doubt, our system’s applicability and benefit for inexperienced examiners needs to be evaluated in further studies.

As stated in our previous report, the electronic guidance systems capability to detect the pelvis position is the key advantage compared to the simple mechanical guidance device recommended by Graf [10].

In our experience, clinical practice has often shown that tilting of the pelvic orientation in the lateral position is often not recognized with the cradle-like positioning aid suggested by Graf.

As a result, despite the use of mechanical aids, undetected tilting of the pelvic orientation often leads to misalignment between the transducer and the pelvis, consequently leading to significantly altered alpha angle measurements. Although experienced examiners are likely to detect and correct tilting errors, our guidance system also enables inexperienced examiners to reduce errors and produce reliable measurements. The electronic guidance system not only reduces the problem of misalignment, but also stores the 3D data for improved documentation of the correct examination technique. Considering the significant reduction in alpha angles in group B, this highlights the clinical relevance of the application of our guidance system, which has the capacity to eliminate errors that might have clinical implications. Consequential clinical implications may be a prolonged or shortened treatment with Pavlik harnesses, which is not only relevant to the socioeconomic aspect, but also improves the newborn’s quality of life by not adding the restriction of a harness. This may lead to an improved predictability regarding treatment duration. Moreover, it should be considered that treatment with a Pavlik harness makes everyday activities such as changing diapers or bathing more difficult for the parents of an affected child. Our results regarding the classification of subtypes according to Graf show that the application of our guidance system results in an increased detection rate of Type II hips by reducing potential tilting errors which consequently suggests that it may influence the treatment in case of “borderline” hips. This finding further highlights the relevance of our novel approach. The increased detection rate of type II hips may result in prolonged treatment or closer follow-up examinations. However, the actual clinical impact needs to be determined in further larger studies with long-time follow-up as our system might be prone to overdiagnosis.

The application of universal ultrasound screening for DDH is still being discussed, with controversy [31]. A review by Kuitunen et al. has shown higher detection rates with universal ultrasound screening; however, the late detection rates and operative treatment rates were shown to be similar to those among clinically and selectively screened newborns [31]. However, the broad regional variety of incidence of DDH, varying screening programs, etc. may be a limitation of this review [31]. Two studies from Austria and Germany, on the contrary, have shown a significant reduction in surgery rates and costs after the introduction of universal ultrasound screening [21,22]. Although universal ultrasound screening may still be up for debate, applications like our guidance system can significantly improve the quality and reproducibility of ultrasound screening, thereby making it applicable even for inexperienced examiners.

The correct alignment between the transducer and the pelvic position has not yet been clearly defined in all planes. The alignment in the frontal and axial planes (roll and pitch angles) is defined as parallel, with a target value of 0° between the transducer and pelvic sensor in these planes; however, no specific target value is established for the sagittal plane (yaw angle) [10]. Further research is needed to define this value for the sagittal plane in order to enhance the guidance system for guidance in the sagittal plane.

The study is mainly limited by the cohort size and the restriction to one examiner. Further studies with a larger sample size as well as multiple examiners will be needed to validate our electronic guidance system. Moreover, the evaluation of inter-observer and intra-observer reliability is a crucial aspect that needs to be addressed in future studies. A potential bias may also occur due to the study protocol in which the hip is first examined conventionally and secondly using our navigation system, since the examiner has already seen the joint before.

However, our results show that the application of our presented electronic guidance system reduces tilting errors, thereby significantly reducing alpha angles, which may have clinical implications. Furthermore, it has the capacity to enable even inexperienced examiners to produce correct and more reproducible measurements, which is a major advantage compared to non-guided examinations. Taking all of these advantages into account, we believe that an implication of our presented electronic guidance system in clinical practice has the potential to improve reproducibility and reliability in the ultrasound screening for DDH. Nevertheless, we clearly state that further larger studies with long-term follow-up are needed to validate it.

## 6. Conclusions

Our electronic guidance system significantly reduced the relative tilt angles between the transducer and the newborn’s pelvis, ensuring optimal positioning of the transducer relative to the pelvis. The correction of tilt errors showed significant effects on Graf sonographic measurements. Our results suggest that this system has the potential to improve reproducibility and reliability in the ultrasound screening for DDH. However, larger clinical studies with long-term follow-up are required to validate this novel application and potential clinical implications resulting from it.

## Figures and Tables

**Table 1:** Overview of relative roll (frontal plane) and pitch angles (axial plane): group A (conventional) and group B (guidance system assisted).

	N	Minimum	Maximum	Mean	Standard Deviation
roll angle (group A)	43	−12.6°	14.3°	0.2°	7.1
pitch angle (group A)	43	−23.8°	32.5°	4.4°	10.9
roll angle (group B)	43	−3.7°	3.0°	0.0°	1.8
pitch angle (group B)	43	−3.2°	4.5°	0.3°	1.8

## Data Availability

The original contributions presented in this study are included in the article. Further inquiries can be directed to the corresponding author.
